# Biodegradation of Petroleum Hydrocarbons by *Bacillus subtilis* BL-27, a Strain with Weak Hydrophobicity

**DOI:** 10.3390/molecules24173021

**Published:** 2019-08-21

**Authors:** Dan Wang, Jiahui Lin, Junzhang Lin, Weidong Wang, Shuang Li

**Affiliations:** 1 College of Biotechnology and Pharmaceutical Engineering, Nanjing Tech University, Nanjing 210009, China; 2 Oil Production Research Institute, Shengli Oil Field Ltd. Co. SinoPEC, Dongying 257000, China

**Keywords:** *Bacillus subtilis*, hydrocarbon-degrading bacterium, biodegradation, cell surface hydrophobicity, surfactant

## Abstract

The biodegradation of petroleum hydrocarbons has many potential applications and has attracted much attention recently. The hydrocarbon-degrading bacterium BL-27 was isolated from petroleum-polluted soil and was compounded with surfactants to improve biodegradation. Its 16S rDNA and *rpoD* gene sequences indicated that it was a strain of *Bacillus subtilis*. Strain BL-27 had extensive adaptability and degradability within a broad range of temperatures (25–50 °C), pH (4.0–10.0) and salinity (0–50 g/L NaCl). Under optimal conditions (45 °C, pH 7.0, 1% NaCl), the strain was able to degrade 65% of crude oil (0.3%, *w*/*v*) within 5 days using GC-MS analysis. Notably, strain BL-27 had weak cell surface hydrophobicity. The adherence rate of BL-27 to *n*-hexadecane was 29.6% with sucrose as carbon source and slightly increased to 33.5% with diesel oil (0.3%, *w*/*v*) as the sole carbon source, indicating that the cell surface of BL-27 is relatively hydrophilic. The strain was tolerant to SDS, Tween 80, surfactin, and rhamnolipids at a concentration of 500 mg/L. The cell surface hydrophobicity reduced more with the addition of surfactants, while the chemical dispersants, SDS (50–100 mg/L) and Tween 80 (200–500 mg/L), significantly increased the strain’s ability to biodegrade, reaching 75–80%. These results indicated that BL-27 has the potential to be used for the bioremediation of hydrocarbon pollutants and could have promising applications in the petrochemical industry.

## 1. Introduction

Petroleum is still an irreplaceable non-renewable energy resource. Increased demand for petroleum products in various industries and daily life may lead to increased oil costs, enhanced oil exploitation, and raised oil pollution [1,2]. Bioremediation of hydrocarbon pollutants and microbial enhanced oil recovery are the two main burning issues of the application of biotechnology in the petroleum industry [3].

Crude oil is a mixture of a variety of simple and complex hydrocarbons which can be degraded by several indigenous microorganisms, each capable of breaking down a specific group of molecules. Numerous research studies concerning biodegradation/bioremediation of hydrocarbon pollutants have been done in recent years. Bacterial sp. of genera *Achromobacter*, *Acinetobacter*, *Arthrobacter*, *Azoarcus*, *Brevibacterium*, *Cellulomonas*, *Corynebacterium*, *Flavobacterium*, *Marinobacter*, *Micrococcus*, *Nocardia*, *Ochrobactrum*, *Pseudomonas*, *Stenotrophomaonas*, and *Vibrio* are reported as hydrocarbon destructors [4]. However, few thermophilic bacteria have been isolated and adapted to biodegradation, with most belonging to the *Bacillaceae*, such as *Bacillus subtilis*, *Geobacillus pallidus*, and *Geobacillus thermodenitrificans* [5,6,7,8]. Cell surface hydrophobicity (CSH) was long considered as a factor to promote adhesion of bacterial water-hydrocarbon interfaces [9]. Usually, higher cell hydrophobicity has been considered to help cells absorb hydrophobic substrates because improving cell surface hydrophobicity is one of the main approaches for facilitating the transport of hydrophobic substrates into cells [10].

A promising method that can enhance hydrocarbon biodegradation effectiveness is the use of surfactant. The synergistic effect of surfactants was recognized to be through two mechanisms: the change of substrate bioavailability for microorganisms and the change of the hydrophobicity of cell surface—allowing substrates to associate more easily with bacterial cells [11,12].

Here, we have reported on the isolation and characterization of the indigenous hydrocarbon-degrading strain BL-27, which was identified as a strain of *Bacillus subtilis* with a weakly hydrophobic cell surface. The effects of different concentrations of chemical dispersants and bio-surfactants on the growth of the bacteria, CSH, and the biodegradation of crude oil were investigated. The aim of this work was to study the potential of this strain of *Bacillus subtilis*, BL-27, to biodegrade crude petroleum oil.

## 2. Results

### 2.1. Isolation and Identification of the Bacterial Strain

**Figure 1:**
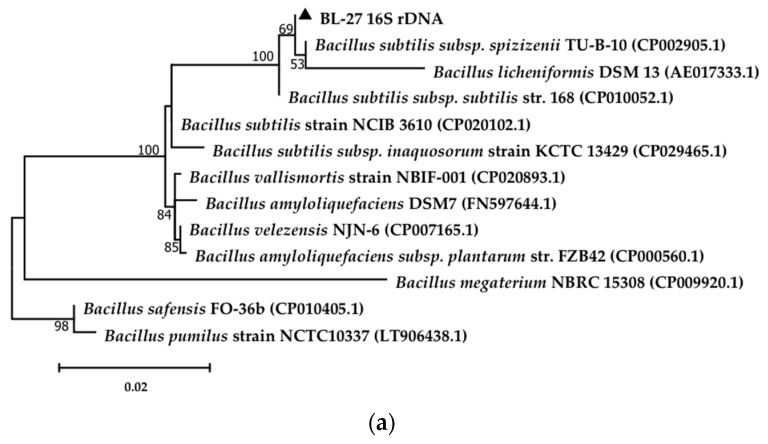

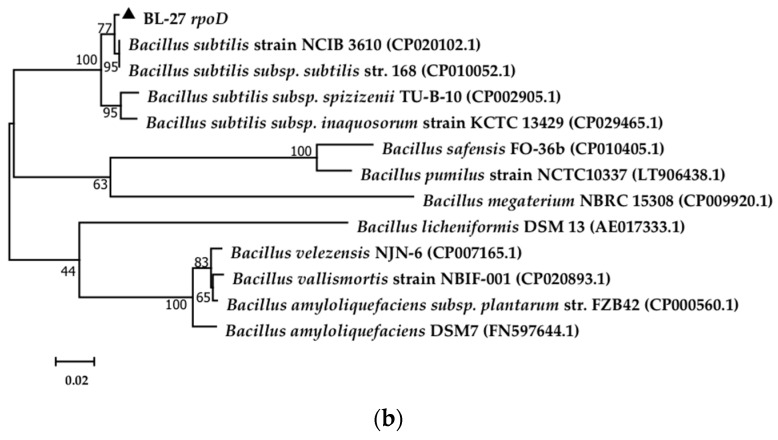
Phylogenetic trees of the strain BL-27 with closely related sequences from the GenBank database; constructed based on the 16S rDNA and *rpoD* gene sequences using the Maximum Likelihood method and GTR model. GenBank accession numbers are shown in parentheses. (**a**) Phylogenetic tree of the strain BL-27 based on 16S rDNA gene sequences; (**b**) Phylogenetic tree of the strain BL-27 based on the *rpoD* gene sequences.

A bacteria designated as BL-27 was isolated from petroleum-polluted soil in the Shengli Oilfield (China) using crude oil as the sole carbon source. To identify the strain, the 16S rDNA and *rpoD* gene sequences were searched against the NCBI database, and model strains with the highest sequence similarity among the aligned sequences were retrieved from the GenBank database. The Maximum Likelihood method and General Time Reversible model were used to create phylogenetic trees of strain BL-27 based on the 16S rDNA and *rpoD* gene (Figure 1). The phylogenetic trees (Figure 1a) shows that the similarity of the 16S rDNA gene sequence between strain BL-27 and *Bacillus subtilis* is 99%, and Figure 1b shows that the similarity of the *rpoD* gene sequence between strain BL-27 and *Bacillus subtilis* NCIB 3610 is 99%. Thus, strain BL-27 was assigned to the genus *Bacillus* sp. and identified as a bona fide strain of *Bacillus subtilis*.

### 2.2. The Effects of Carbon Source, Temperature, pH and Salinity on Strain Growth and Biodegradation of Crude Oil

The *n*-alkanes (C7–C25) and several other hydrocarbons were used as the sole carbon source to investigate the utilization of hydrocarbons by strain BL-27. As shown in Table 1, strain BL-27 could use medium- to long chain *n*-alkanes (C17–25), ethanol, acetonitrile, and diesel oil as the optimum substrates, and the biomass (OD_600_) could reach 0.7–1.3. The short-to-medium-chain *n*-alkanes (C7–C16), cyclohexane, xylene, naphthalene, phenanthrene, trichloromethane, ethyl acetate, and liquid paraffin could be slightly utilized by the strain, and the range of the biomass was 0.1–0.7. BL-27 could not use phenol, styrene, and isoamylol for growth. This experiment laid the foundation for subsequent application of hydrophobic carbon sources.

**Figure 2:**
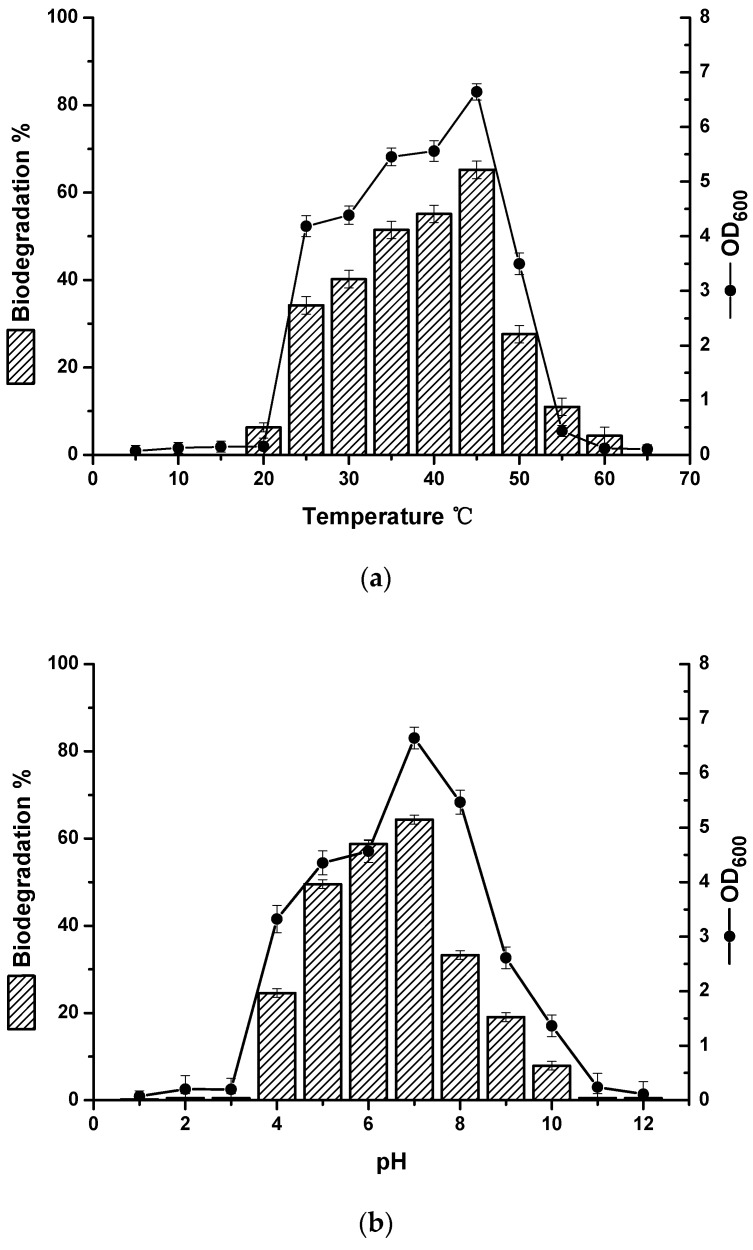

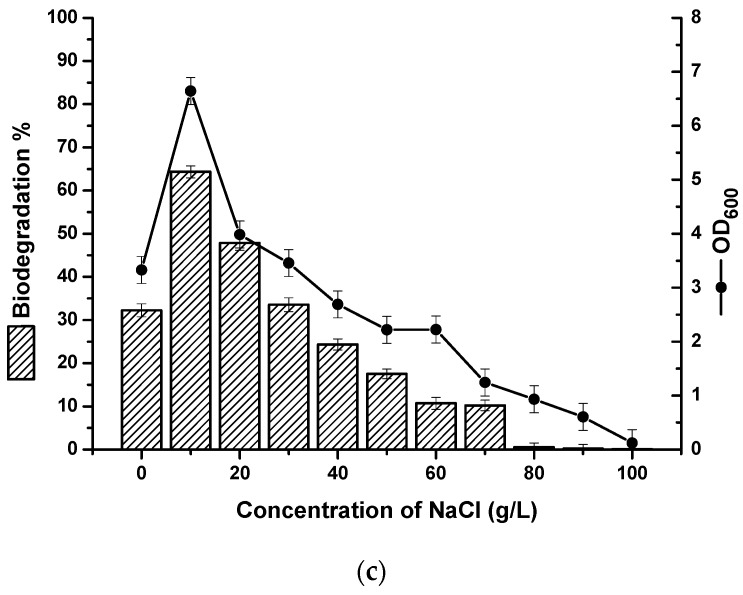
Effects of (**a**) temperature (5–65 °C) at a pH of 7.0 and salinity of 10 g/L; (**b**) pH (1.0–12.0), at a temperature of 45 °C, and salinity of 10 g/L; and (**c**) salinity (0–100 g/L) at a temperature of 45 °C and pH of 7.0 on the cell growth of *B. subtilis* strain BL-27 in LB medium and biodegradation of crude oil in MSM medium at 150 rpm by the strain BL-27 (6%, *v*/*v*).

The optimum temperature for the growth of strain BL-27 strain and degradation of crude oil was 45 °C, with an adaptive range of temperatures from 25 to 50 °C (Figure 2a).

The optimal initial pH for strain BL-27 was shown in Figure 2b. The optimal pH for the growth and degradation of crude oil was 7.0. However, strain BL-27 grew well at initial pH values of 4.0–10.0, showing a wide tolerance to initial pH values, which makes it suitable for most hydrocarbon-polluted environments.

The tolerance to initial NaCl concentration was determined, as shown in Figure 2c. The optimum initial concentration of NaCl was 10 g/L. As the NaCl concentration was increased, the biomass concentration and biodegradation showed an obvious decrease. The salinity tolerance range of strain BL-27 was 0–90 g/L and obvious degradation of crude oil was observed at salinities from 0–50 g/L.

Taken together, the optimum conditions for the growth of the strain were a temperature of 45 °C, initial pH of 7.0, and initial salinity of 10 g/L. The maximum crude oil degradation rate was about 65% under these conditions. The results showed that the growth of strain BL-27 positively correlated with the degradation of crude oil, which agreed with a study of a similar strain by Palanisamy et al. (2014) [13]. It, therefore, has the potential to be widely used in hydrocarbon degradation as a strain with good resistance to acid, alkali, and salt.

### 2.3. GC-MS Analysis of Residual Alkanes after Biodegradation

The residual oil extracted after biodegradation was separated, and its components were determined quantitatively by GC-MS analysis. Peaks of *n*-alkanes from C11 to C30 were clearly distinguishable in the GC chromatogram (data not shown), and their concentrations were calculated using naphthalene as an internal standard. Short-chain *n*-alkanes of C8–C10 were almost lost 100% after 5 days incubation in the control flasks, which indicated that evaporative losses might result in the removal of these components [14].

**Figure 3:**
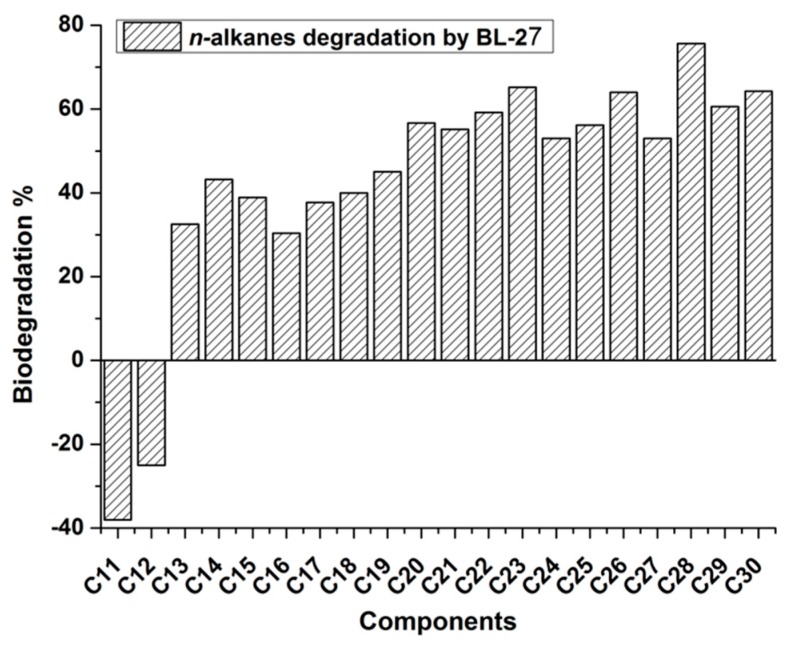
The variation of *n*-alkane components of crude oil after biodegradation by strain BL-27.

Figure 3 showed the component variation of crude oil after degradation. The results indicated that 30–80% of the *n*-alkanes (C13–C30) were degraded by strain BL-27 within 5 days. The increase of C11 and C12 was most likely due to the accumulation of degraded metabolites of long-chain *n*-alkanes.

### 2.4. Analysis of Cell Surface Hydrophobicity (CSH)

**Figure 4:**
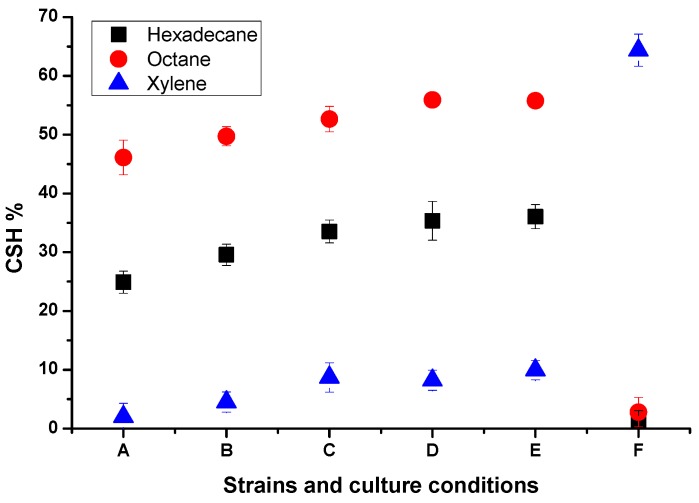
Cell surface hydrophobicity (CSH) of strain BL-27 in different media. Legend meanings: (A: BL-27 in LB medium; B: BL-27 in MSM medium with sucrose; C: the 1st generation of BL-27 in MSM medium with diesel oil; D: the 2nd generation of BL-27 in MSM medium with diesel oil; E: the 3rd generation of BL-27 in MSM medium with diesel oil; F: *E. coli* BL21 in LB medium.).

The BATH test [15] was used to evaluate the cell surface hydrophobicity. Higher BATH values indicate a more hydrophobic cell surface. Generally, a cell surface with a value of more than 80% is considered highly hydrophobic, and a CSH% of less than 30% indicates that the surface of bacterial cells is highly hydrophilic [16]. The results obtained from strain BL-27 (Figure 4) showed that the CSH was dependent on the substrate used for the reaction. When BL-27 was grown to the logarithmic phase (OD_600_ = 1) in LB medium, the values of CSH with hexadecane, octane, and xylene were 24.91, 46.13, and 2.03%, respectively, which indicated that the order of adsorption of hydrophobic substrates by the cells was octane > hexadecane > xylene, and verified that the cells had different hydrophobicity toward different substrates. The accuracy of the data was controlled using *E. coli* BL21. As shown in Figure 4, the hydrophobicity of strain BL-27 cells increased when cultured in MSM medium, as compared with LB medium. When strain BL-27 was grown in MSM supplemented with sucrose and diesel oil, the values of CSH toward hexadecane slightly increased between the first and second generations of subcultured bacteria with diesel oil, from 29.58% to 33.52% and 35.33%, respectively. Thus, the BL-27 cells still had weak hydrophobicity in MSM medium with hydrophobic substrates as the sole carbon source. Compared with hydrophilic substrates, adding hydrophobic substrates might improve the hydrophobicity of the strain’s cells to adapt to the hydrophobic conditions, which was also observed by Liu T et al. (2012) [17]. However, the CSH of strain BL-27 remained practically unchanged (from 35.33% to 36.06%) during its growth on diesel oil from the second to the third generation. Thus, the CSH of the strain exhibited an apparent limit, above which the CSH would not change further. However, limiting factors could not be elucidated within the scope of this study.

### 2.5. The Effects of Different Surfactants on Growth, CSH and Crude Oil Degradation by Strain BL-27

We hypothesize that surface active agents (chemical surfactant or biosurfactant) increasing the bioavailability of hydrocarbon and changing the CSH of cells could result in enhanced growth and degradation of crude oil by strain BL-27. The tolerance of strain BL-27 towards several chemical dispersants and bio-surfactants was investigated to assess its applicability in their presence. Seven individual surfactants were selected for testing. As shown in Table 2, strain BL-27 was sensitive to cationic surfactants—cetyltrimethylammonium bromide (CTAB) and tetradecyl trimethyl ammonium bromide (TTAB)—but was tolerant to other kinds of surfactants. Notably, Tween 80 and SDS had no significant effects on biomass accumulation at 500 mg/L; while biosurfactant surfactin and rhamnolipids showed slight inhibition of growth. Triton X-100 had a significant inhibitory effect on cell growth.

Based on the results of surfactant tolerance, four surfactants, including two chemical dispersants (SDS and Tween 80) and two bio-surfactants (surfactin and rhamnolipids), were selected to study the CSH of BL-27 at high and low concentrations, respectively. It had been reported that surfactants had different effects on CSH of various hydrocarbon degrading microorganisms; the change was highly bacterium and surfactant dependent [18]. As shown in Table 2, all surfactants lowered the CSH of strain BL-27 at low or high concentration. According to Z. Zhao et al. (2011) [19], one possible reason is that the hydrophobic part of the cell surface interacts with the hydrophobic moiety of the surfactant, exposing the hydrophilic part of the cell and the surfactant to the aqueous phase and reducing the CSH.

**Figure 5:**
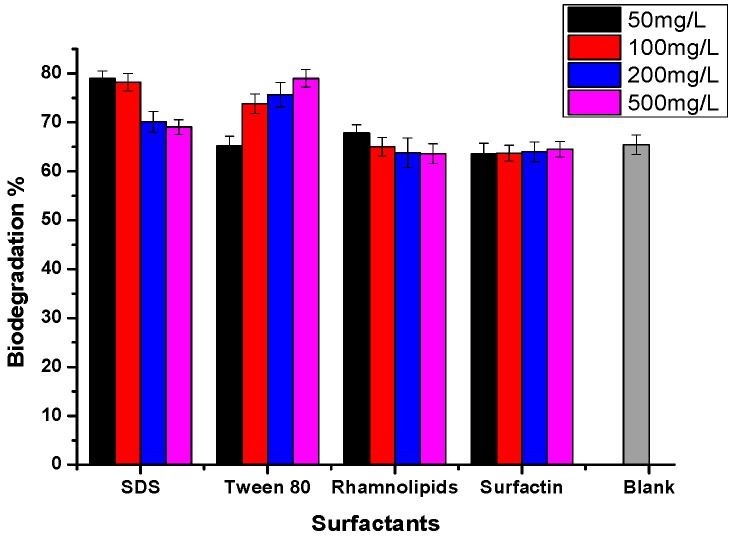
Crude oil biodegradation by strain BL-27 in the presence of surfactants at various concentrations. Blank means the absence of surfactants (0 mg/L).

The effects of the four surfactants on crude oil biodegradation were shown in Figure 5. The biodegradation of crude oil without surfactant was about 65%, and obvious enhancement was observed with additional SDS and Tween 80. The highest degradation of crude oil, reaching 75–80%, was observed with SDS at concentrations of 50–100 mg/L as well as Tween 80 at concentrations of 200–500 mg/L, while the biodegradation rate was almost unaffected with the bio-surfactants surfactin and rhamnolipids.

## 3. Discussion

The strain BL-27 had good adaptability and degradability within a broad range of temperature (25–50 °C), which enables its application in areas with wide temperature fluctuations between day and night, such as bioremediation of crude oil contaminated soil, biochemical treatment of oilfield wastewaters, etc. Microorganisms that degrade hydrocarbons were commonly reported to be mesophilic bacteria isolated from the environment [20,21,22] or thermophilic bacteria isolated from special high-temperature reservoirs [23,24]. However, there have been fewer studies on hydrocarbon-degrading thermophilic bacteria. Furthermore, thermophilic hydrocarbon-degrading bacteria have great potential for improving oil recovery from specific locations, particularly medium–high temperature reservoirs, and could be widely applied in the process of oil washing and soil remediation. In addition, hydrocarbon pollution in the marine environment is regarded as an important international issue [25]. However, the high salinity of seawater (30–40 g/L), poor nutrient content, and a lack of attachment surfaces make microbial hydrocarbon degradation challenging. The strain BL-27 was able to adapt to a wide range of temperatures and degrade 25–35% of crude oil in 5 days at 30–40 g/L salinity. Therefore, it could be used to further test the degradation of hydrocarbons in seawater.

Table 1 indicated that the optimal substrates for BL-27 were *n*-alkanes, diesel oil, acetonitrile, and ethanol. Generally, *n*-alkanes with carbon numbers less than or equal to 21 are considered short-chain alkanes, and those with carbon numbers greater than or equal to 22 are long-chain alkanes [26]. When crude oil was used as the sole substrate, the following conclusions could be drawn according to the variation of residual *n*-alkane components. Notably, the preferential degradation of long-chain *n*-alkanes was observed in strain BL-27, as indicated by the *n*-alkane ΣC21−/ΣC22+ ratio, calculated as (sum of *n*-alkanes with a carbon number less than or equal to 21)/(sum of *n*-alkanes with a carbon number great than or equal to 22) [27], which increased from 0.96 to 1.12. Similarly, the relative abundance of short-chain C21 and C22 in the total amount of *n*-alkanes was compared to that of long-chain C28 and C29 to determine the length of carbon chains that the strains preferentially degrade [28,29]. The (C21 + C22)/(C28 + C29) ratio increased from 1.33 to 2.43 after degradation. Therefore, both the ΣC21−/ΣC22+ and (C21 + C22)/(C28 + C29) ratios indicated that strain BL-27 preferentially degraded long-chain alkanes.

The substrate selectivity and CSH of crude oil-degrading strains are quite different, as shown in Table 3. Among them, the reported thermophilic *Geobacillus stearothermophilus* A-2, *Bacillus subtilis YB7*, *Bacillus methylotrophicus* USTBa, and *Pseudomonas sp.* BP10 are all stronger hydrophobic strains, while the studied *Bacillus subtilis* BL-27 is weakly hydrophobic and displayed good degradation of crude oil. Compared with the strongly hydrophobic strains, strain BL-27 had better salt tolerance (10–30 g/L). Whether the concentration of NaCl affects the CSH of the strain remains to be studied further.

Recently, more and more research reports have been published about the influence of surfactants on biodegradation or bioremediation. Both positive and no/negative effects of the influence of surfactants were observed. The microbial toxicity of (bio)surfactants is a possible cause of biodegradation inhibition; also, a possible increased toxicity of hydrophobic substrates was presented due to their increased (pseudo)solubility [33]. In our work, CTAB and TTAB exhibited serious toxic effects on BL-27; the biosurfactants rhamnolipid and surfactin had no effects on biodegradation, while SDS and Tween 80, at their optimal concentrations, could significantly enhance the biodegradation of crude oil by strain BL-27. Overall, as shown in Table 4, microorganisms had specific responses to surfactants amended for the biodegradation of petroleum hydrocarbons. The characteristics of microbes, the composition of petroleum hydrocarbons, and the interaction mechanisms of surfactants are diverse, resulting in different effects

Therefore, screening strains and selecting surfactant, especially the optimization of surfactant concentration, could play a crucial role in the application of bacterial biodegradation or bioremediation of petroleum hydrocarbons.

## 4. Materials and Methods

### 4.1. Crude Oil, Diesel Oil, Chemicals and Culture Media

The crude and diesel oil used for biodegradation experiments were provided by Sinopec Yangzi Petrochemical Co., Ltd., (Nanjing, China). All other chemicals were purchased from local commercial sources and were of analytical grade.

The Luria-Bertani (LB) medium was composed of (g/L): yeast extract 5.0, peptone 10.0, NaCl 10.0; agar plates were made by adding 2% (*w*/*v*) agar into the medium. The basic mineral salt medium (MSM) with crude- or diesel oil as the sole carbon source was composed of (g/L): NH_4_NO_3_ 3.0, KH_2_PO_4_ 1.5, K_2_HPO_4_ 1.5, NaCl 10.0, MgSO_4_ 0.1, FeSO_4_ 0.01., and CaCl_2_ 0.01. The pH was adjusted to 7.0–7.2 by adding 1.0 M NaOH, and the medium was sterilized by autoclaving at 121 °C for 20 min.

### 4.2. Isolation and Identification of Bacteria

Soil samples for isolation of potential crude oil degrading bacteria were collected in triplicate in the areas affected by oil contamination in the Shengli Oilfield (Dongying, China) and below the surface (0–10 cm) and were transported aseptically at 4 °C to the laboratory. A sample comprising approximately 5 g of soil was used to inoculate a 250 mL flask with 100 mL of MSM medium with 0.3% (*w*/*v*) crude oil as the sole carbon source. The flask was shaken at 150 rpm at 45 °C for 7 days, after which 2 mL of the culture was transferred to fresh MSM medium with crude oil, and the culture was continued for another 7 days. The resulting enrichment culture was diluted and spread on MSM agar plates containing crude oil as the sole carbon source. The resulting bacterial colonies were further purified by streaking on LB agar plates and grown at 45 °C for 12 h. The final strain was designated as BL-27 and stored at −80 °C in 30% (*w*/*v*) sterile glycerol.

The 16S rDNA gene sequence had some limitations because of its low resolution for bacteria with close genetic relationships. It could only distinguish bacteria at the genetic level. Some RNA polymerase genes, such as *rpoD*, had shown great superiority in identifying closely related strains [38]. In this paper, 16S rDNA and *rpoD* gene sequences were used to identify the strain. Genomic DNA was isolated from a pure culture of the strain using the Genomic DNA Extraction Kit (Bioteke Biotechnology Co., Ltd., Beijing, China), and polymerase chain reaction (PCR) was performed to amplify the 16S rDNA and *rpoD* gene. The 16S rDNA of strain BL-27 was amplified using the universal primers 27F (5′-AGAGTTTGATCCTGGCTCAG-3′) and 1492R (5′-GGTTACCTTGTTACGACTT-3′) for bacterial fragments (≈1500 bp). The sequence of the *rpoD* gene (≈1000 bp) was determined using the primers BS-F (5′-ATGGCTGATAAACAAACCCACGAGA-3′) and BS-R (5′-TTATTCGAGGAAATCTTTCAAACGT-3′), which were designed from conserved regions of the *rpoD* nucleotide sequence of members of the *Bacillus* group [39,40]. The DNA sequence was double-checked by sequencing both strands and searched against GenBank using the BLAST algorithm. A phylogenetic tree was constructed using the Maximum Likelihood method and General Time Reversible model in MEGA 7.0 software [41] by bootstrapping 1000 times.

### 4.3. Carbon Source Utilization

The experiment was designed to determine the utilization of different hydrocarbon substrates as the sole carbon sources by strain BL-27. The microbial enrichment culture was inoculated at 6% (*v*/*v*) into individual flasks containing 100 mL of MSM medium with individual *n*-alkanes (C7-C25), cyclohexane, xylene, liquid paraffin, diesel oil, phenol, naphthalene, phenanthrene, styrene, ethanol, acetonitrile, trichloromethane, isoamylol, and ethyl acetate, respectively. These compounds were added to the MSM medium at 0.1% (*w*/*v* for solids, *v*/*v* for liquid chemicals) and incubated at 30 °C and 150 rpm for 3 days. MSM medium without bacteria was used as a sterile control. All experiments were performed in triplicate. For each sample, the cells were harvested by centrifugation at 12,000× *g* for 5 min, washed twice with sterile distilled water, and resuspended in the same volume of sterile distilled water. Growth of the strain was measured by the increase of optical density (OD) at 600 nm using a conventional spectrophotometer.

### 4.4. Optimization of Environmental Parameters for the Growth of Strain BL-27 and Degradation of Crude Oil

Various environmental factors were considered in the experimental design, including temperature, pH, and salinity, which were optimized by conducting batch experiments in LB medium for strain growth and in MSM medium containing 0.3% (*w*/*v*) crude oil for the degradation of crude oil. These experiments were conducted at different temperatures of 5–65 °C, pH 1.0–12.0 (adjusted using 1 M NaOH or 1 M HCl), and salinity (the concentration of NaCl) of 0–10.0% (*w*/*v*). An aliquot comprising 100 mL of liquid culture medium was added to 250 mL of flasks. After sterilization, 6.0% (*v*/*v*) of BL-27 bacteria seed culture was added to the flasks and incubated on the shaker at 150 rpm for 24 h. For each sample, the cells were separated by centrifugation at 12,000 × *g* for 5 min, washed twice with sterile distilled water, and resuspended in the same volume of sterile distilled water. The growth of strain BL-27 in LB medium was determined by measuring the optical density (OD) at a wavelength of 600 nm by using a visible spectrophotometer. After 5 days, the amount of residual crude oil left in MSM medium was determined by extracting with an equal volume of petroleum ether and analyzing the organic phase at 225 nm using a UV spectrophotometer.

Quantitative analysis of the *n*-alkane*s* in crude oil was conducted on an Agilent 6890–5975 C gas chromatography mass spectrometer (GC-MS; Agilent, Palo Alto, CA, USA) equipped with an HP-5MS capillary silica column (60 m × 0.25 mm × 0.25 μm). The degradation of alkanes by strain BL-27 was studied by growing in MSM medium containing crude oil as the sole carbon source at 45 °C and 150 rpm for 5 days. Non-inoculated sterile medium served as a negative control. All experiments were conducted in triplicate. After incubation, the culture was extracted with equal volumes of petroleum ether. The extracts were dehydrated with anhydrous Na_2_SO_4_, dried at room temperature and used as the residual crude oil sample, which was fractionated into saturated hydrocarbons, aromatic hydrocarbons, non-hydrocarbons, and asphaltenes [42] on a silica gel column with neutral aluminum oxide (100–200 mesh) according to the Chinese Standard SY/T 5779-2008 [30]. The alkanes were re-dissolved into *n*-hexane with quantitative naphthalene as internal standard. The sample injection volume was 2 μL. The GC settings were as follows: injector temperature, 250 °C; detector temperature, 250 °C; carrier gas (He 99.999%); flow rate, 1.0 mL/min; split ratio, 2:1. The oven temperature began at 50 °C for 1 min and increased to 120 °C at 20 °C/min, before increasing to 250 °C by 4 °C/min, where it remained for 30 min [43]. Mass spectrometry settings were as follows: electron impact, electron energy 70 eV; filament current 100 μA; multiplier voltage 1.2 kV; selective ion monitoring (SIM) mode [44].

### 4.5. Analysis of Cell Surface Hydrophobicity

Cell surface hydrophobicity (CSH) was assessed using bacterial adhesion to hydrocarbons (BATH), as described by Rosenberg et al. (1980) [45], with minor modifications as follows: Strain BL-27 was grown to the logarithmic phase (OD_600_ = 1) in 100 mL of LB medium at 45 °C and 150 rpm. The bacterial cells were harvested by centrifugation at 8000× *g* for 10 min, washed three times with 0.16 mol/L NaCl, resuspended in sterile distilled water and adjusted to an OD_600_ of 0.5–0.6. Subsequently, 4 mL of the bacterial suspension in a round-bottomed test tube was mixed with 1 mL of *n*-hexadecane, *n*-octane, xylene, diesel oil, or crude oil, respectively, vortexed for 2 min and put aside for 15 min to facilitate phase separation at room temperature. Next, the OD_600_ of the aqueous phase was measured. A bacterial suspension without any organic phase, sterile distilled water with different organic phases, and *E. coli* BL21 were used as controls. Each experiment was repeated three times, and cell surface hydrophobicity (CSH) was calculated using the formula: [(A_0_ − A_1_)/A_0_] × 100%, where A_0_ is the initial bacterial cell concentration in the aqueous phase, and A_1_ is the cell concentration in the aqueous phase after adsorption [46].

### 4.6. Cell Surface Hydrophobicity Test of Cells Cultured with Different Carbon Sources

In order to determine whether hydrocarbon adsorption of the cells changes after cultivation with different carbon sources, a cell surface hydrophobicity (CSH) test was used. The seed cell suspension (6%, *v*/*v*) was transferred to 100 mL of either LB medium, MSM medium containing 20 g/L sucrose, or MSM medium with 3 g/L diesel oil as the sole carbon source, and the strain in MSM medium with diesel oil was subcultured to the third generation. After the growth of the strain to logarithmic phase (OD_600_ = 1), and CSH was measured as described above. Each experiment was done in triplicate, and cell hydrophobicity was expressed as the mean value of three flasks.

### 4.7. The Effect of Surfactants on Strain Growth, CSH and the Biodegradation of Crude Oil

The experiment was designed to determine the tolerance of strain BL-27 for different surfactants. The seed culture was used at 6% (*v*/*v*) to inoculate 100 mL of LB medium with the addition of either Tween 80, rhamnolipids, surfactin, Triton X-100, sodium dodecyl sulfate (SDS), cetyltrimethylammonium bromide (CTAB), or tetradecyl trimethyl ammonium bromide (TTAB). The surfactants were added to LB medium at concentrations of 50, 100, 200, and 500 mg/L, and the cells incubated at 45 °C for 24 h at 150 rpm. LB medium without surfactant was used as a positive control. All experiments were performed in triplicate. For each sample, the cells were separated by centrifugation at 12,000× *g* for 5 min, washed twice with sterile distilled water, and resuspended in the same volume of sterile distilled water. Growth of the strain was measured by the increase of OD_600_.

The strain BL-27 was cultured in LB media containing either Tween 80, SDS, rhamnolipids, or surfactin at two concentrations (50 and 200 mg/L) and grown to the logarithmic phase (OD_600_ = 1) for the CSH experiments. The control group was LB medium without surfactants. Each sample was measured in triplicates, and the results were expressed as the mean values of three flasks.

Two sets of oil-biodegradation experiments were carried out in this study. The first set investigated the impact of different surfactants on crude oil biodegradation, with Tween 80, SDS, rhamnolipids, and surfactin as models. The second set of experiments examined the effect of surfactant concentrations at 50, 100, 200, and 500 mg/L. The biodegradation process was carried out in 250 mL flasks containing 100 mL of MSM medium with 0.3% (*w*/*v*) crude oil as the sole carbon source at 45 °C and 150 rpm for 5 days. Non-inoculated and surfactant-free flasks were used as controls, and each experiment was done in triplicate. After the reaction, the remaining oil in the flask was extracted as described above and analyzed using the UV spectrophotometer at 225 nm.

## 5. Conclusions

*Bacillus subtilis* BL-27, isolated from hydrocarbon contaminated sites, was a hydrocarbon-degrading bacterium. The strain had weak hydrophobicity, which could be improved by hydrophobic substrates to some extent. Under the optimized conditions, encompassing 45 °C, pH of 7.0, and 10 g/L of NaCl, the strain could degrade 65% of crude oil after 5 days, whereby long-chain alkanes were preferentially degraded. The CSH of the strain was decreased with the addition of surfactants, but the crude oil degradation rate was significantly increased to 75–80% by the chemical dispersants, SDS and Tween 80, at their optimal concentrations. BL-27 has the potential to be used for the bioremediation of hydrocarbon pollutants (3 g/L) and could have promising applications in the petrochemical industry.

## Figures and Tables

**Table 1:** Range of carbon sources utilized by strain BL-27 in MSM medium.

Carbon Source	Growth ^a, b^	Carbon Source	Growth ^a, b^
C7	+	C23	++
C8	+	C24	++
C9	+	C25	++
C10	+	Cyclohexane	+
C11	+	Xylene	+
C12	+	Phenol	-
C13	+	Naphthalene	+
C14	+	Phenanthrene	+
C15	+	Styrene	-
C16	+	Ethanol	++
C17	++	Acetonitrile	++
C18	++	Trichloromethane	+
C19	++	Isoamylol	-
C20	++	Ethyl acetate	+
C21	++	Liquid paraffin	+
C22	++	Diesel oil	++

^a^ Growth of the strain was assessed by measuring the OD600. ^b^ (++), obvious growth (0.7 ≤ OD600 < 1.3); (+), weak growth (0.1 ≤ OD600 < 0.7); (-), no growth (OD600 < 0.1).

**Table 2:** The effect of surfactants at various concentrations on the growth and CSH of strain BL-27.

Surfactant Concentration (mg/L)	Relative Growth %					CSH * %		
	50	100	200	500	0	50	200	0
SDS	103 ± 1.2	102 ± 1.5	100 ± 1.4	100 ± 1.3	100	16.4 ± 1.9	18.7 ± 2.3	24.9 ± 1.9
Tween 80	99 ± 1.6	101 ± 1.6	101 ± 1.7	103 ± 1.8	100	22.6 ± 1.8	12.6 ± 1.6	24.9 ± 1.9
Rhamnolipids	97 ± 1.6	85 ± 2.0	83 ± 1.3	85 ± 2.1	100	16.9 ± 1.4	9.5 ± 2.1	24.9 ± 1.9
Surfactin	89 ± 1.6	87 ± 1.3	88 ± 1.1	85 ± 1.6	100	22.1 ± 2.1	19.4 ± 1.7	24.9 ± 1.9
Triton X-100	48 ± 2.2	41 ± 4.2	37 ± 3.9	27 ± 3.1	100	ND	ND	ND
CTAB	6.2 ± 1.3	6.2 ± 1.2	5.7 ± 1.3	5.3 ± 1.3	100	ND	ND	ND
TTAB	3.9 ± 1.3	3.9 ± 1.2	3.5 ± 1.3	3.3 ± 1.4	100	ND	ND	ND

ND: not detected. * CSH toward *n*-hexadecane.

**Table 3:** The characteristics of some crude oil-degrading strains.

Strains	Temperature (°C)	Salinity (g/L)	Preferential Degradation Components	Biodegradation Rate (g/L/d)	Substrates of CSH	CSH %	Reference
*Geobacillus stearothermophilus* A-2	60	0	C22-C33	0.164	hexadecane	83.9% ^a^	[30]
*Bacillus subtilis* YB7	50	0.5	*n*-alkanes	0.633	hexadecane	72–95% ^b^	[5]
*Bacillus methylotrophicus* USTBa	35	0	*n*-alkanes	1.314	crude oil	62.0% ^b^	[31]
*Pseudomonas* sp. BP10	35	0	C10-C28	0.404	crude oil	70.0% ^b^	[32]
*Bacillus subtilis* BL-27	45	10	C17-C30	0.387	hexadecane	24.9% ^a^ 33.5% ^b^	This work

^a^ Cultured in LB medium. ^b^ Cultured in MSM medium with diesel oil.

**Table 4:** The effects of surfactants on CSH and biodegradation of hydrocarbons.

Surfactant	Concentration	Strains	CSH %			Degradation %			Reference
			Substrates	Without Surfactant	With Surfactant	Substrates	Without Surfactant	With Surfactant	
Rhamnolipids	600 mg/L	*Pseudomonas* LSH-7	crude oil	-	-	crude oil	77%	87%	[34]
Rhamnolipids	400 mg/L	*Bacillus subtilis* BUM	phenanthrene	23%	25%	phenanthrene	82%	32%	[35]
Rhamnolipids	120 mg/L	*Aeromonas hydrofila*	diesel oil	7%	12%	diesel oil	58%	60%	[36]
Tween 80	1000 mg/L	*Polyporus* sp. S133	phenanthrene	-	-	phenanthrene	30%	47%	[37]
	5000 mg/L							72%	
	10000 mg/L							61%	
SDS	50 mg/L	*Bacillus subtilis* BL-27	*n*-hexadecane	25%	16%	crude oil	65%	79%	This work
	200 mg/L				19%			70%	
Tween 80	50 mg/L				23%			65%	
	200 mg/L				13%			76%	
Rhamnolipids	50 mg/L				17%			68%	
	200 mg/L				10%			64%	
Surfactin	50 mg/L				22%			64%	
	200 mg/L				19%			64%	
